# No Effect of Low‐Dose Aspirin Versus Placebo as Add‐On Treatment in Bipolar Disorder—Results From a Randomised Controlled Trial (the A‐Bipolar RCT)

**DOI:** 10.1111/acps.70055

**Published:** 2025-11-30

**Authors:** Caroline Fussing Bruun, Helle B. Krogh, Jeff Zarp, Julie Ravneberg Stokholm, Julie Lyng Forman, Kamilla Woznica Miskowiak, Annamaria Giraldi, Maj Vinberg, Maria Faurholt‐Jepsen, Lars Vedel Kessing

**Affiliations:** ^1^ Copenhagen Affective Disorder Research Centre (CADIC), Psychiatric Centre Copenhagen Copenhagen University Hospital ‐ Mental Health Services CPH Copenhagen Denmark; ^2^ Department of Clinical Medicine, Faculty of Health and Medical Sciences University of Copenhagen Copenhagen Denmark; ^3^ Sexological Clinic, Mental Health Centre Copenhagen Copenhagen University Hospital ‐ Mental Health Services CPH Copenhagen Denmark; ^4^ Section of Biostatistics, Department of Public Health, Faculty of Health and Medical Sciences University of Copenhagen Copenhagen Denmark; ^5^ NEAD Centre, Department of Psychology, Faculty of Social Sciences University of Copenhagen Copenhagen Denmark; ^6^ The Early Multimodular Prevention and Intervention Research Institution (EMPIRI), Psychiatric Centre North Zealand Copenhagen University Hospital ‐ Mental Health Services CPH Hillerød Denmark

**Keywords:** aspirin, bipolar disorder, mood instability, randomised controlled trials

## Abstract

**Introduction:**

Robust evidence associates immunoinflammatory dysfunction and bipolar disorder (BD), with immune dysregulation present in patients newly diagnosed with BD. This suggests that anti‐inflammatory agents, like low‐dose aspirin (LDA), might be repurposed in the treatment of early‐stage BD. Building on pharmacoepidemiologic and meta‐analytic evidence, we conducted the first randomised controlled trial (RCT) testing the effects of add‐on LDA in patients with newly diagnosed BD. We hypothesised that add‐on treatment with LDA would reduce mood instability (MI), activity instability (AI), and depression severity.

**Methods:**

In this parallel group, triple‐blind, superiority RCT, patients newly diagnosed with BD, recruited from a public outpatient mood disorder clinic in the Capital Region of Denmark, were randomised 1:1 to 150 mg acetylsalicylic acid or placebo by an independent third party. The primary outcome was average MI at 6‐month follow‐up, assessed by the Root Mean Square of Successive Differences (RMSSDs) method. Secondary outcomes included average AI, assessed by the RMSSD method, and change in depressive symptoms, assessed with the 6‐item Hamilton Depression Rating Scale, at 6 months. Tertiary outcomes included sleep variability, cognition, manic symptoms and questionnaires at 6 months.

**Results:**

Two hundred fifty patients were randomised from January 20, 2022, to May 30, 2024, with the last follow‐up on November 18, 2024. Analyses included data from 240 patients (120 received placebo; 120 LDA), with a mean of 108 (SD = 57) daily mood registrations. The estimated treatment difference for MI was 0.022 (95% CI: −0.056 to 0.1, *p* = 0.58) for LDA compared to placebo, indicating no clinically relevant difference. No beneficial effects of LDA were found across secondary or tertiary outcomes. Methodologically, randomisation and blinding were successful, and serum‐thromboxane B2 levels confirmed high adherence to LDA.

**Conclusions:**

Low‐dose aspirin did not demonstrate any benefit on MI or on secondary or tertiary outcomes in patients with newly diagnosed BD.

**Clinical Registration:**

Clinicaltrials.gov registration number: NCT05035316

## Summary

- Significant Outcomes
  ○ In this methodologically stringent, well‐powered randomised controlled trial investigating the effects of add‐on treatment with low‐dose aspirin versus placebo in patients with newly diagnosed bipolar disorder, we found no beneficial effects on the primary outcome, patient‐reported daily mood instability.
  ○ No beneficial effects were observed across secondary outcomes and tertiary outcomes.
  ○ Blinding was maintained, adherence to low‐dose aspirin was high (as confirmed by serum‐thromboxane B_2_) and dropout and missing data were balanced, enhancing internal validity and reducing type 2 error risk.
- Limitations
  ○ Our sample consisted of newly diagnosed (within 2 years) patients with few medical comorbidities and a relatively low baseline symptom burden, possibly limiting generalisability to patients with more symptomatic or progressed illness.

## 1 Introduction

Bipolar disorder (BD) is a severe mental illness (SMI) with a lifetime prevalence of 1%–2% [1], characterised by phasic alterations in mood and activity. Despite available pharmacological and non‐pharmacological treatments, many patients with BD experience residual symptoms and disease progression [2, 3]. Thus, new treatments for BD, ideally targeting the underlying biological mechanisms, are needed.

Meta‐analytic evidence and recent studies of BD in general [4, 5, 6] and of newly onset BD specifically [7, 8], indicate a possible link between immunoinflammatory dysfunction and BD [9, 10, 11, 12]. Although it remains uncertain whether these findings reflect causal mechanisms, confounding influences, or consequences of the illness itself, it has led to interest in the repurposing of anti‐inflammatory agents [9, 10, 11, 12]. Specifically, low‐dose acetylsalicylic acid (LDA) has been proposed [13], and a recent meta‐analysis by our group [14] found that add‐on aspirin treatment was more effective than placebo in reducing depressive symptoms in patients with SMI (standardised mean difference (SMD) = −0.71, 95% confidence interval (CI): −1.40 to −0.02, 6 studies, I^2^ = 87%, *p* = 0.04), with a 36% higher treatment response in patients with major depressive disorder (relative risk [RR] = 1.36; 95% CI: 1.07 to 1.72, 2 studies, I^2^ = 0%). However, the certainty of the evidence was *very low*, and clinical heterogeneity, along with methodological concerns in the only four available RCTs with patients with BD were a considerable limitation. While pharmacoepidemiologic evidence suggests that LDA may benefit patients with BD [15, 16], no clinical trial has definitively shown if LDA has a role in the treatment.

Many patients with BD continuously experience inter‐episodic daily mood swings. Although subsyndromal, these day‐to‐day mood fluctuations are clinically relevant, as they are associated with decreased quality of life, functioning, and increased subjective stress levels [17], as well as risk of relapse and hospitalisation [18, 19]. The daily mood swings can be quantified by the measure of mood instability (MI), calculated from time series of daily self‐reported, smartphone‐collected mood ratings, using the root mean square of successive differences (RMSSD) method [20]. Mood instability measures variability in mood over time, allowing for the detection of early, clinically relevant improvements. This may increase statistical power and sensitivity in RCTs. In this way, MI represents an alternative to traditional outcomes like hospitalisations or relapse [20]. Further, MI exhibits trait‐like characteristics [21] and may represent a distinct phenotype with biological underpinnings that could be targeted with LDA.

The aim of this RCT was to assess the efficacy and tolerability of adding LDA in the treatment of BD, irrespective of clinical phase, using MI as the primary outcome measure. We hypothesised that adding LDA versus placebo to standard treatment would reduce (1) MI; and (2) activity instability (AI) and severity of depression, assessed with the 6‐item Hamilton Depression Rating Scale (HDRS‐6) [22, 23].

## 2 Materials and Methods

See the study protocol [24] and the Supplement for details on the methods.

### 2.1 Study Design

We conducted a two‐arm, triple‐blind, parallel‐group, superiority RCT including 250 patients with newly diagnosed BD with patient‐reported MI as the primary outcome. The study was registered on Clinicaltrials.gov (NCT05035316) and approved by the Danish Research Ethics Committee (H‐21014515) and the Danish Data Protection Agency Capital Region of Denmark (P‐2021‐576). The study was conducted in accordance with the principles of the Helsinki Declaration and monitored by the Good Clinical Practice (GCP) unit of Copenhagen University, following international guidelines [25]. The results are reported following the Consolidated Standards of Reporting Trials (CONSORT) guidelines [26].

### 2.2 Participants and Recruitment

Participants were recruited from two public outpatient mood disorder clinics (The Copenhagen Affective Disorder Clinics [Copenhagen and Hilleroed]) that treat the majority (> 80%) of all patients newly diagnosed with BD (within the past 2 years) in the Capital Region of Denmark [27]. Eligibility was evaluated by medical doctors, who also obtained informed consent. Patients aged 18–65 with a primary diagnosis of BD were enrolled from January 20, 2022, to May 30, 2024, with the last follow‐up on November 18, 2024. Exclusion criteria were conditions or treatments contraindicating LDA. See the Supplement and protocol [24] for details.

### 2.3 Randomisation and Blinding

Participants were randomised in a 1:1 ratio to LDA or placebo using block randomisation with randomly varying block sizes of 6, 8, 10 and 20. The Hospital Pharmacy of the Capital Region of Denmark generated the allocation sequence using the Sealed Envelope software (https://www.sealedenvelope.com/). Low‐dose aspirin and placebo were encapsulated and identical in appearance and pre‐packed in sealed, consecutively numbered, identically labelled plastic bins. When a patient had consented to participate, a randomisation number and a corresponding bin were irreversibly assigned by the investigators.

### 2.4 Treatments and Visits

The participants received 150 mg acetylsalicylic acid (LDA) or placebo (calcium) orally once daily as add‐on treatment. Adherence was evaluated at the follow‐up visits by interviews and pill counts. Serum‐thromboxane (TX) B_2_ was measured at 3 and 6 months in a random subset of ~30% of participants. Because LDA irreversibly inhibits cyclooxygenase‐1, blocking the conversion of arachidonic acid to TXA_2_ and reducing its stable metabolite, TXB_2_, TXB_2_ serves as an objective marker for LDA adherence [28].

Mood registration adherence was monitored weekly, with reminders for fewer than five entries per week.

Participation entailed three visits, at baseline, after 3 months and 6 months. Participants that agreed to continue for a full year had a final visit after 12 months. See the protocol [24] for a detailed description of visits and assessments.

### 2.5 Outcomes

The primary outcome was *MI*, calculated from daily, patient‐rated mood assessments and evaluated by the mean monthly RMSSD. The secondary outcomes were *activity instability*, calculated from daily, self‐reported activity ratings and evaluated by the mean monthly RMSSD [29] and *depressive symptoms*, assessed with the Hamilton Depression Rating Scale‐6 items (HDRS‐6) [22, 23].

Tertiary outcomes included clinical ratings (*manic symptoms*, assessed with the Young Mania Rating Scale [YMRS]; *functioning*, assessed with the Functioning Assessment Short Test [FAST] [30]; *cognition*, assessed with the Screen for Cognitive Impairment in Psychiatry [SCIP] tool) [31]; patient‐reported outcomes using validated questionnaires (*quality of life*, assessed with the World Health Organization [WHO] Quality of Life‐BREF questionnaire [32]; *subjective stress levels*, assessed with Cohen's Perceived stress scale [33]; *physical activity*, assessed with the International Physical Activity Questionnaire [34]; *sleep quality*, assessed with the Pittsburgh Sleep Quality Index [35]); and *variability in self‐reported hours of sleep*, assessed by the RMSSD method [36].

Exploratory biomarker measures included a panel of 16 cytokines and hair cortisol. Results from these analyses will be presented in a future publication.

Clinical ratings were conducted by trained investigators with an intraclass correlation coefficient of 0.899.

### 2.6 Statistical Analyses

In newly diagnosed patients with BD, MI varies on a scale from close to 0 to 10 with an average MI of 1.8 (95% CI: 1.12 to 1.24) and a standard error (SE) of 0.7 [21]. Thus, to detect a clinically relevant decrease of 0.3 with treatment compared with placebo with a power of 80% and a significance level of 0.05, a total of 172 patients needed to be randomised. To accommodate attrition and non‐compliance, we included 250 patients in the study.

For each study participant, monthly MI during follow‐up was calculated using the RMSSD method when there were mood ratings for two consecutive days. The mean difference in average MI during 6‐month follow‐up was evaluated using a linear mixed model (LMM) including treatment group, time and the interaction between them as fixed effects and an unstructured covariance pattern to account for repeated measurements on each participant. For exploratory purposes, monthly differences in mean MI between LDA and placebo with 95% CIs were also reported. Missing data were handled implicitly by maximum likelihood estimation in the linear mixed model.

Activity instability and hours of sleep were analysed in the same way as MI. The remaining secondary and tertiary outcomes were evaluated using a constrained LMM with inherent baseline adjustment including follow‐up time and the constrained time–treatment interaction as fixed effects and an unstructured covariance pattern. The *p*‐values from the secondary and tertiary analyses were adjusted using the method of Benjamini and Hochberg [37].

The primary and secondary analyses were repeated in the per‐protocol population, defined as participants reporting taking the trial medication ≥ 50% of the time and completing ≥ 30 days of mood assessments.

Exploratory subgroup analyses were conducted for the most symptomatic participants, defined as baseline HDRS‐6 or YMRS scores > median; participants in an affective phase at baseline, defined as HDRS‐17 scores ≥ 14 or YMRS scores ≥ 14, as well as by sex (men/women); age (≥ median/< median); BD subtype (type 1 or 2); presence of a first‐degree relative with affective disorder; and those with 100% investigator‐rated adherence to trial medication at the 6‐month follow‐up. Further, post hoc exploratory analyses included correlations between MI and psychometric ratings (HDRS‐17, HDRS‐6, YMRS and FAST).

All analyses were carried out according to a pre‐specified statistical analysis plan [24] and conducted in RStudio v.2024.04.2 using the LMMstar package [38] for linear mixed model analyses.

## 3 Results

### 3.1 Participant Flow

**FIGURE 1:**
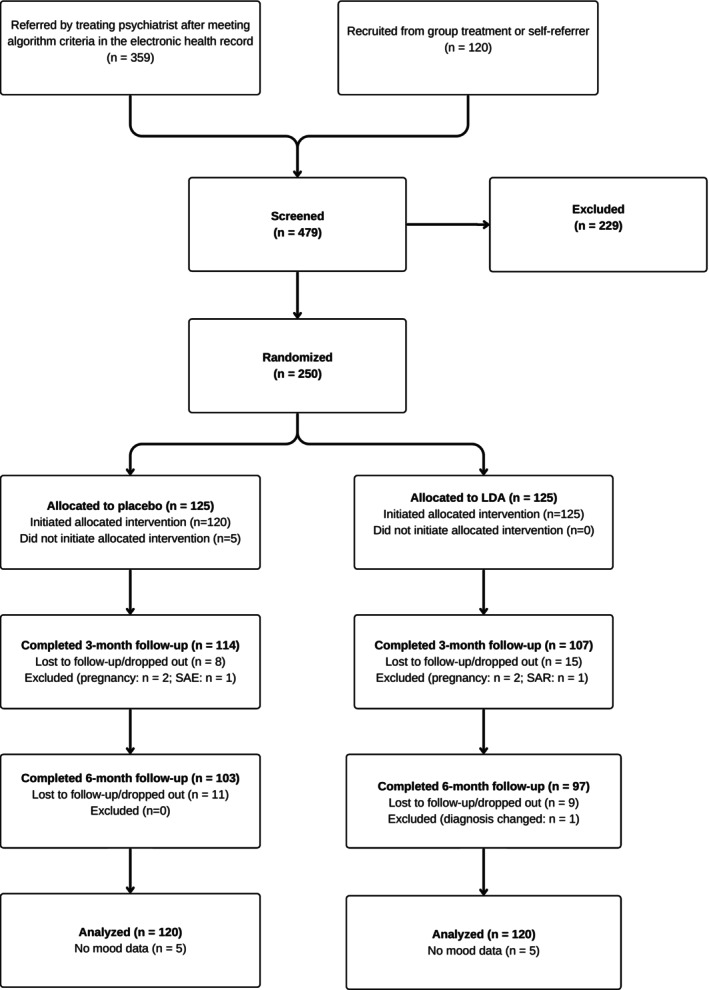
CONSORT flow diagram.

Figure 1 is the CONSORT flow diagram for the study. A total of 479 patients were assessed for eligibility, of which 229 were excluded (66% male, 34% female); median age 28 years (interquartile range [IQR]: 24–35). The main reasons were too demanding trial requirements, lack of motivation, or unwillingness to take trial medication (Table S1). Of the 250 randomised participants, 221 completed the 3‐month and 200 the 6‐month follow‐up. At the 6‐month follow‐up, 87 volunteered to continue for an additional 6 months, with 83 completing the 12‐month follow‐up. Further, 83 did not wish to continue after the 6‐month period, while the remaining 80 participants were not offered this option due to time restrictions. Post‐randomisation dropout rates and reasons for dropout or exclusion were similar across groups (Tables S2, S3).

### 3.2 Baseline Characteristics

Baseline demographic and clinical variables, stratified by treatment group, are presented in Tables 1 and S4–S7. The sample consisted of 250 participants with a median age of 29.5 years (IQR: 32.6–38), 58% females and 43% males, and 48% had BD type 1 and 52% had type 2.

**TABLE 1:** Baseline demographic and clinical characteristics of included patients with bipolar disorder.

	PBO (*n* = 125)		LDA (*n* = 125)	
Demographics				
Age (Mdn, Q1; Q3)	29	25; 40	30	25; 37
Females (*n*, %)	72	57.6	73	58.4
Cis gender (*n*, %)	115	92.0	113	90.4
Married/registered partnership or stable relationship (*n*, %)	69	55.2	57	46.0
Years of education^a^, M (SD)	14.8	2.7	14.9	2.5
Full‐time employed or student (*n*, %)	73	58	69	55
Number of long‐term^b^ sick leaves due to BD (Mdn, Q1; Q3)	2	1; 4	2	0; 3
Clinical characteristics				
Bipolar type 1 (*n*, %)	65	52.0	55	44.0
Age at BD illness onset (Mdn, Q1; Q3)	17	15; 20	19	16; 22
Duration of BD illness (years) (Mdn, Q1; Q3)	11	6; 20	10	5; 16
Depressive episodes (Mdn, Q1; Q3)	7	5; 17.5	7	4; 15
Hypomanic episodes (Mdn, Q1; Q3)	8	3; 20	6	3; 18
Manic episodes (Mdn, Q1; Q3)	0	0; 2	0	0; 1
Mixed episodes (Mdn, Q1; Q3)	0	0; 1	0	0; 0
≥ 1 suicide attempt(s) (*n*, %)	28	22.4	22	17.6
≥ 1 hospitalisation(s) (*n*, %)	51	40.8	39	31.2
≥ 1 psychotic episode (*n*, %)	36	28.8	33	26.4
≥ 1st degree relative with mental illness (ICD‐10 criteria) (*n*, %)	165	66	78	62.4
HDRS‐17 total score (Mdn, Q1; Q3)	8	4; 13	9	6; 14
HDRS‐6 total score (Mdn, Q1; Q3)	4	2; 7	5	3; 8
YMRS total score (Mdn, Q1; Q3)	4	2; 7	4	2; 8
Psychotropic medication				
Antidepressants (non‐SSRIs) (*n*, %)	4	3.2	7	5.6
Quetiapine (*n*, %)	57	46.0	55	44.0
Lamotrigene (*n* %)	65	52.0	66	52.8
Lithium (*n*, %)	57	45.6	61	48.8
Benzodiazepines or Z‐hypnotics PN (*n*, %)	8	6.4	9	7.2
Functioning				
FAST total score (Mdn, Q1; Q3)	17	10; 24	16	10; 28
Cognition				
SCIP total score (Mdn, Q1; Q3)	79	70; 87	77	71; 84
Questionnaire scores				
Childhood trauma questionnaire score (Mdn, Q1; Q3)	40.5	32; 48	41.5	31; 52
Overall quality of life (M, SD)	3.5	0.9	3.3	0.8
Cohen's Perceived Stress Scale (M, SD)	21	6	22	6
International Physical Activity Questionnaire—short form, MET‐min/week (Mdn, Q1; Q3)	2474	1244; 3885	2253	1218; 4284
Pittsburgh Sleep Quality Index (Mdn, Q1; Q3)	8	6; 11	9	7; 12

Abbreviations: FAST: functioning assessment short test; HDRS‐17: Hamilton Depression Rating Scale 17‐item version; HDRS‐6: Hamilton Depression Rating Scale 6‐item version; ICD, international classification of diseases; LDA, low‐dose aspirin; M, mean; Mdn, median; MET: metabolic equivalent task; PBO, placebo; PN: pro re nata/as needed; Q1, 25th percentile; Q3, 75th percentile; SCIP: screen for cognitive impairment in psychiatry; SD, standard deviation; SSRIs: selective serotonin reuptake inhibitors; YMRS: Young Mania Rating Scale.

^a^ Completed years, starting with primary school.

^b^ Defined as sick leave ≥ 2 weeks.

### 3.3 Adherence to Study Medication and Mood Assessments

Self‐reported compliance was high and equal between the groups (Table S8). Analyses of TXB_2_ showed highly reduced levels in the LDA group compared to placebo, supporting LDA adherence throughout the trial (Figure S1).

On average, participants completed mood ratings 108 (SD = 57) out of 182 days, with similar frequency across groups (Figure S2). Self‐reported reasons for missingness of ratings did not differ between groups (Table S9).

### 3.4 Blinding Assessment

Results from the blinding assessment showed that participants' and investigators' allocation guess at the last follow‐up were not significantly different from chance (participants: 52.1% correct guesses, 95% CI: 0.45 to 0.59, *p* = 0.61; investigators: 51.1% correct guesses (95% CI: 0.44 to 0.58, *p* = 0.83)), indicating that the blinding was maintained throughout the trial. Two participants were unblinded and excluded for safety reasons: in one case, a 46‐year‐old male participant had a suspected intracerebral haemorrhage after a head trauma (placebo); in another, a 25‐year‐old female participant experienced prolonged bleeding after a medical abortion (LDA).

### 3.5 Treatment Effect

#### 3.5.1 Primary Outcome

The primary analyses included 240 participants (120 in the placebo group and 120 in the LDA group), as 10 participants (5 in each group) never started using the Monsenso app. The estimated treatment difference (ETD) in average MI during the 6‐month follow‐up was 0.022 (95% CI: −0.056 to 0.1, *p* = 0.58) for LDA compared to placebo, indicating no clinically relevant difference (Table 2). This persisted in the analysis at the 12‐month follow‐up (Table 4). Analyses of monthly MI showed decreasing trends in means over time for both treatment groups with no significant differences between treatments (Figure S3 and Table S10). We found no significant differences in the per‐protocol population (Table 2).

**TABLE 2:** Intention‐to‐treat and per‐protocol analyses using linear mixed models: Low‐dose aspirin versus placebo as add‐on treatment in patients with bipolar disorder—primary and secondary outcomes at 6‐month follow‐up.

Outcome	Population	PBO		LDA		ETD	95% CI		*p*	Adj. *p*
		M	SD	M	SD		LL	UL		
**Primary outcome**										
**Mood instability**, PBO: *n* = 120; LDA: *n* = 120	ITT	0.487	0.324	0.520	0.305	0.022	−0.056	0.1	0.58	—
**Mood instability**, PBO: *n* = 80; LDA: *n* = 77	PP	0.502	0.291	0.495	0.273	−0.007	−0.096	0.082	0.881	0.962
**Secondary outcomes**										
**Activity instability**, PBO: *n* = 120; LDA: *n* = 119	ITT	1.27	0.484	1.37	0.492	0.111	−0.019	0.24	0.094	0.094
**Activity instability**, PBO: *n* = 80; LDA: *n* = 77	PP	1.29	0.486	1.40	0.476	0.111	−0.041	0.264	0.152	0.608
**Depressive symptoms** (HDRS‐6), PBO: *n* = 105; LDA: *n* = 97	ITT	−1.22	4.35	−0.608	5.17	1.195	0.23	2.16	0.016	0.032
**Depressive symptoms (HDRS‐6)**, PBO: *n* = 80; LDA: *n* = 77	PP	4.38	2.70	5.22	2.37	1.44	0.331	2.55	0.011	0.264

Abbreviations: 95% CI, 95% confidence interval; Adj., adjusted; ETD, estimated treatment difference from linear mixed model analyses, relative to placebo group; HDRS‐6, Hamilton Depression Rating Scale 6‐item version; ITT, intention‐to‐treat; LDA, low‐dose aspirin; LL, lower limit; M, mean; PBO, placebo; PP, per‐protocol; SD, standard deviation; UL, upper limit.

#### 3.5.2 Secondary and Tertiary Outcomes

The LDA group had an *increase* in HDRS‐6 from baseline to the 6‐month follow‐up compared to placebo (ETD = 1.195, 95% CI: 0.23 to 2.16, *p* = 0.016, *adjusted p =* 0.032) (Table 3). This was replicated in the per‐protocol population (ETD = 1.44, 95% CI: 0.331 to 2.55, *p* = 0.011, *adjusted p* = 0.360). However, from baseline to the 12‐month follow‐up, the ETD was −0.882 (95% CI: −2.447 to 0.683, *p* = 0.266, *adjusted p* = 0.558) (Table 4).

**TABLE 3:** Results of linear mixed model analyses comparing the effect of a low‐dose aspirin versus placebo as add‐on treatment in patients with bipolar disorder—tertiary outcomes at 6‐month follow‐up.

Outcome	PBO		LDA		ETD*	95% CI		*p*	Adj. *p*
	M	SD	M	SD		LL	UL		
**Change in manic symptoms** (YMRS), PBO: *n* = 103; LDA: *n* = 96	−0.825	5.32	−0.0104	5.98	0.639	−0.613	1.924	0.328	0.656
**Change in functioning** (FAST), PBO: *n* = 103; LDA: *n* = 96	−3.85	10.2	−2.20	12.1	2.069	−0.619	4.758	0.131	0.608
**Change in cognition** (SCIP), PBO: *n* = 64; LDA: *n* = 63	2.12	8.27	3.63	7.30	0.4691	−2.024	2.961	0.710	0.962
**Change in overall quality of life** (WHO QoL‐BREF), PBO: *n* = 100; LDA: *n* = 86	0.18	0.881	0.151	0.819	−0.121	−0.313	0.072	0.218	0.654
**Change in stress** (PSS), PBO: *n* = 99; LDA: *n* = 86	−2.16	5.29	−2.79	5.79	−0.15	−1.587	1.288	0.837	0.962
**Change in physical activity** (IPAQ), PBO: *n* = 98; LDA: *n* = 85	−68.7	3557	−88.9	4037	302	−756	1360	0.574	0.962
**Change in sleep quality** (PSQI), PBO: *n* = 99; LDA: *n* = 85	−0.848	2.97	−1.07	3.64	0.004	−0.8	0.809	0.992	0.992
**Change in sleep variability**, PBO: *n* = 120; LDA: *n* = 120	1.74	0.684	1.73	0.650	−0.026	−0.197	0.144	0.761	0.962

Abbreviations: 95% CI, 95% confidence interval; Adj., adjusted; ETD, estimated treatment difference from linear mixed model analyses, relative to placebo group; FAST, functioning assessment short test; IPAQ, International Physical Activity; LDA, low‐dose aspirin; LL, lower limit; M, mean; PBO, placebo; PSQI, Pittsburgh Sleep Quality Index; PSS, Cohen's Perceived Stress Scale; SCIP, screen for cognitive impairment in psychiatry; SD, standard deviation; UL, upper limit; WHO QoL‐BREF, WHO Quality of Life‐BREF questionnaire; YMRS, Young Mania Rating Scale.

**TABLE 4:** Results of exploratory linear mixed model analyses comparing the effect of a low‐dose aspirin versus placebo as add‐on treatment in patients with bipolar disorder—all outcomes at 12‐month follow‐up.

Outcome	PBO		LDA		ETD	95% CI		*p*	Adj. *p*
	M	SD	M	SD		LL	UL		
**Mood instability**, PBO: *n* = 121^a^; LDA: *n* = 120	0.461	0.309	0.494	0.292	0.003	−0.007	0.076	0.934	0.975
**Activity instability**, PBO: *n* = 121^a^; LDA: *n* = 119	1.26	0.477	1.35	0.497	0.093	−0.054	0.241	0.214	0.654
**Change in depressive symptoms** (HDRS‐6), PBO: *n* = 42; LDA: *n* = 41	−0.286	5.35	−1.17	4.53	−0.882	−2.447	0.683	0.266	0.656
**Change in manic symptoms** (YMRS), PBO: *n* = 42; LDA: *n* = 41	0.238	5.40	−0.976	4.63	−1.034	−2.924	0.856	0.279	0.656
**Change in functioning** (FAST), PBO: *n* = 42; LDA: *n* = 41	−0.833	13.9	−3.88	10.7	−3.826	−8.176	0.524	0.084	0.608
**Change in overall quality of life** (WHO QoL‐BREF), PBO: *n* = 39; LDA: *n* = 37	0.179	0.914	0.216	0.917	0.124	−0.199	0.447	0.448	0.827
**Change in stress** (PSS), PBO: *n* = 40; LDA: *n* = 38	−3.35	7.40	−3.63	4.76	−0.185	−2.654	2.284	0.882	0.962
**Change in physical activity** (IPAQ), PBO: *n* = 41; LDA: *n* = 37	−256	3032	188	2922	−253	−1845	1339	0.752	0.962
**Change in sleep quality** (PSQI), PBO: *n* = 40; LDA: *n* = 37	−1.23	2.82	−0.108	3.36	1.214	0.036	2.392	0.044	0.528
**Change in sleep variability**, PBO: *n* = 121; LDA: *n* = 120	1.72	0.696	1.72	0.656	0.018	−0.178	0.214	0.859	0.962

Abbreviations: 95% CI, 95% confidence interval; Adj., adjusted; ETD, estimated treatment difference from linear mixed model analyses, relative to placebo group; FAST, functioning assessment short test; IPAQ, International Physical Activity; LDA, low‐dose aspirin; LL, lower limit; M, mean; PBO, placebo; PSQI, Pittsburgh Sleep Quality Index; PSS, Cohen's Perceived Stress Scale; SCIP, screen for cognitive impairment in psychiatry; SD, standard deviation; UL, upper limit; WHO QoL‐BREF, WHO Quality of Life‐BREF questionnaire; YMRS, Young Mania Rating Scale.

^a^ *n* = 121 as 1 participant started using the Monsenso app after the 6‐month follow‐up.

We found no difference in AI between the groups in the full sample for the 6‐month or 12‐month follow‐up, or in the per‐protocol subsample (Tables 2 and 4).

No differences between the groups were found across all tertiary outcomes (Tables 3 and 4).

### 3.6 Exploratory Subgroup Analyses

For forest plots of the exploratory subgroup analyses, see Figures S11–S17.

For MI, exploratory subgroup analyses from baseline to the 6‐month follow‐up indicated no effect of LDA on MI in any subgroup. The ETDs were small and non‐significant with narrow CIs including zero, except for participants with YMRS ≥ 14, where the CI was wider (*n* = 19). Similarly, at 12 months, analyses of the subgroups with baseline YMRS > median, baseline HDRS‐17 ≥ 14, and baseline YMRS ≥ 14 yielded imprecise estimates, with lower CI limits suggesting potential clinical relevance.

For AI, the LDA group experienced an *increase* compared to placebo at the 6‐month follow‐up in the subgroups with baseline age < median and BD type 2. However, the effects did not reproduce in the 12‐month analyses.

For depressive symptoms, participants who received LDA showed a decrease in HDRS‐6 scores compared to placebo at 6 months (ETD = −1.207, 95% CI: −2.355 to −0.058). Conversely, other subgroups (male, younger, high adherence) showed *increased* HDRS‐6 at 6 months, with wide CIs indicating imprecision but potential clinical worsening at the upper limits. At 12 months, the male sex (ETD = −2.606, 95% CI: −4.902 to −0.309) and older age (ETD = −2.268, 95% CI: −4.551 to 0.016) subgroups demonstrated reductions in HDRS‐6, a pattern opposite to the 6‐month findings.

Finally, MI during the first 30 days correlated with baseline scores on HDRS‐17 (*r* = 0.371, 95% CI: 0.255 to 0.477), HDRS‐6 (*r* = 0.303, 95% CI: 0.182 to 0.416), YMRS (*r* = 0.184, 95% CI: 0.057 to 0.306) and FAST (*r* = 0.240, 95% CI: 0.115 to 0.358).

### 3.7 Adverse Effects

Overall, the intervention was well‐tolerated with few adverse reactions (ARs) (6 in the placebo group; 9 in the LDA group). Four participants (two from each group) withdrew due to gastrointestinal side effects. One serious adverse reaction (SAR) was recorded (see ‘blinding assessment’). There was no significant difference in the occurrence of ARs, Serious Adverse Events (SAEs), or SARs between allocation groups. Specifically, no differences were observed for gastrointestinal ARs, including reflux or pyrosis, nausea or vomiting, and increased bleeding tendency/bruising. No sudden unexpected serious adverse reactions were recorded (Tables S11–S13).

### 3.8 Missing Data

Participants reported depressive symptoms as the second most frequent reason (30%) for missing mood assessments, with reasons unrelated to BD (e.g., busy work schedule, simply forgetting) as the most frequent (40%). However, missingness (both the frequency and the reasons) was non‐differential between groups (Figure S2 and Table S9), with no differences across baseline variables when comparing participants who completed the 6‐month follow‐up with non‐completers (Table S14). Similarly, participants who met per‐protocol criteria did not differ from participants who did not (Table S15).

## 4 Discussion

This first large triple‐blind RCT revealed no beneficial effects of LDA on the primary outcome, mood instability (MI), the secondary outcomes, activity instability (AI) and depression severity, or the tertiary outcomes. For the primary outcome, the findings were consistent for the 12‐month follow‐up, in the per‐protocol sample, and monthly treatment effect analyses. Unexpectedly, we observed a slight *worsening* in HDRS‐6 scores in the LDA group at the 6‐month follow‐up, which persisted in the per‐protocol subgroup and among the 100% adherent. However, this was not observed for the 12‐month follow‐up.

This was the first RCT to use MI as the primary outcome, and the theoretical possibility that this could account for the lack of efficacy should be acknowledged; yet the absence of any signal across the multiple outcomes argues convincingly against this explanation. Further, no clear patterns of subgroup effects were identified in the post hoc exploratory subgroup analyses. Although post hoc subgroup analyses in RCTs are often underpowered, most effect estimates at the 6‐month follow‐up were close to zero with narrow confidence intervals, suggesting that no clinically significant treatment effects were missed. The sporadic observed differences may reflect true effects or statistical fluctuations due to multiple comparisons and small samples. Especially for the depressive symptoms outcome (HDRS‐6) and for participants with the most manic symptoms at baseline, the estimates were imprecise, likely due to small subgroup sample sizes.

In the referenced meta‐analyses [14], aspirin reduced depressive symptoms compared to placebo in patients with SMIs. However, incorporating data from the present RCT resulted in no significant difference in depressive symptoms between placebo (*n* = 291) and aspirin (*n* = 274) (SMD −0.55, 95% CI: −1.18 to 0.09, 7 studies, *I* ^*2*^ = 91%, *p* = 0.09). Further, updated BD subgroup analyses showed no effect on manic (SMD 0.10, 95% CI: −0.11 to 0.31, 5 studies, I^2^ = 0%, *p* = 0.35) or depressive symptoms (SMD −0.09, 95% CI: −0.55 to 0.36, 5 studies, I^2^ = 58%, *p* = 0.69) comparing aspirin (*n* = 164) to placebo (*n* = 179).

In the Youth Depression Alleviation with Antiinflammatory Agents (YoDA‐A) trial, Berk et al. found no effect of adjunctive aspirin (100 mg/day) on depressive symptoms in patients aged 15–25 with moderate‐to‐severe unipolar depression [39]. Taken together, findings from the YoDA‐A and A‐bipolar trials, both testing adjunctive LDA in the early stage of mood disorders, have failed to replicate the signals observed in independent two‐pharmacoepidemiologic studies [15, 16]. Notably, the doses (150 vs. 100 mg) and duration (24 vs. 12 weeks) in the two RCTs varied, and the optimal dose and treatment duration remain uncertain. Further, the pathophysiological processes that we aim to target may begin prior to the onset of clinical symptoms and diagnosis.

Strengths of the study include that we conducted a methodologically rigorous, well‐powered RCT with a sensitive, clinically meaningful and well‐validated primary outcome measure [20] that correlated with scores at inclusion on HDRS‐17, HDRS‐6, YMRS and FAST, strengthening the internal validity of the trial. Further, we included state‐of‐the‐art psychiatric evaluation, observer‐based as well as patient‐reported outcomes measures, and up to 12 months of follow‐up. Blinding was maintained, adherence to low‐dose aspirin was high (as confirmed by serum‐TXB_2_), and dropout and missing data were balanced, enhancing internal validity and reducing type 2 error risk.

Limitations include that our sample consisted of newly diagnosed patients with relatively few affective symptoms at baseline, a rather high level of functioning, and few medical comorbidities, possibly limiting generalisability to BD patients with more symptoms or progressed illness. The focus on newly diagnosed patients was intentional, as we aimed to explore early prevention in BD. Further, MI occurs beyond syndromic affective phases, and our analyses confirmed that the participants experienced a significant reduction in MI over time. This may reflect either the sensitivity of MI to detect subtle improvements in the study or reflect regression to the mean. Our sample did not permit an investigation of the potential moderating effect of pre‐existing cardiovascular (CVD) risk factors; building on the shared inflammatory component in BD and CVD [40], it is an interesting consideration whether a subgroup with pre‐existing CVD risk factors would benefit from LDA.

The rationale for the 150 mg dose was based on evidence from pharmacoepidemiologic studies that identified doses ranging from 75–150 mg to 30–80 mg, respectively [15, 16], as potentially beneficial in BD. This was not found for higher doses, which are furthermore known to carry a non‐negligible bleeding risk. Our meta‐analysis [14] showed no indication of differential effects between low‐ (≤ 150 mg) and high‐dose (> 150 mg) aspirin. Yet, we cannot rule out that higher doses of aspirin might be effective.

In conclusion, LDA showed no benefit on our primary, secondary, or tertiary outcomes in patients with newly diagnosed BD. No clear patterns of subgroup effects were identified in post hoc exploratory subgroup analyses. While the results robustly rule out a rationale for the broad clinical use of LDA in early‐stage BD, it remains unexplored whether LDA may have a potential beneficial effect in subgroups of patients with BD and comorbid physical risk factors such as obesity, hypertension and hyperlipidemia.

## Author Contributions

**Caroline Fussing Bruun:** writing – original draft, writing – review and editing, funding acquisition, project administration, investigation, data curation. **Helle B. Krogh:** investigation, writing – review and editing, data curation. **Jeff Zarp:** investigation, writing – review and editing, data curation. **Julie Ravneberg Stokholm:** investigation, writing – review and editing, data curation. **Julie Lyng Forman:** methodology (statistical analyses), supervision, writing – review and editing. **Kamilla Woznica Miskowiak:** methodology (cognition), writing – review and editing. **Annamaria Giraldi:** writing – review and editing. **Maj Vinberg:** conceptualisation, methodology, supervision, writing – review and editing. **Maria Faurholt‐Jepsen:** conceptualisation, methodology (mood instability), supervision, writing – review and editing. **Lars Vedel Kessing:** primary investigator and guarantor, conceptualisation, methodology, supervision, funding acquisition, writing – review and editing.

## Funding

This study was supported by the Research Fund of the Mental Health Services ─ Capital Region of Denmark (November 2020), Svend Andersen Fonden (03‐10‐2022), Lægeforeningens Forskningsfond (2022‐0011), Overlæge Dr. Med. Einar Geert‐Jørgensen og Hustrus Legat (16‐11‐2022), and Ivan Nielsens fond for personer med specielle lidelser (07018018). The funding sources had no role in the study design, conduction of the study, or interpretation of the data.

## Conflicts of Interest

C.F.B., J.R.S., K.W.M., and J.L.F. have no competing interests to declare. H.B.K. and J.Z. have, within the last 3 years, received honoraria from Lundbeck Pharma A/S. L.V.K. has within the preceding 3 years been a consultant for Lundbeck and Teva. M.V. has within the last 3 years served as consultant for Lundbeck, Eli Lilly and Janssen/Cilag. M.F.‐J. has within the last 3 years served as consultant Janssen/Cilag.

## Supporting information


**Data S1:** Supporting Information.

## Data Availability

The data that support the findings of this study are available on request from the corresponding author. The data are not publicly available due to privacy or ethical restrictions.
