# Supplementary material for: No Effect of Low‐Dose Aspirin Versus Placebo as Add‐On Treatment in Bipolar Disorder—Results From a Randomised Controlled Trial (the A‐Bipolar RCT)
Source: Acta Psychiatr Scand. 2025 Nov 30;153(3):214–24. doi: 10.1111/acps.70055 (PMC12861576; doi:10.1111/acps.70055)
Supplement: Supplementary file 1 — Data S1: Supporting Information. [file ACPS-153-214-s001.docx]

Effects of low-dose aspirin versus placebo as add-on treatment in bipolar disorder - results from a controlled trial (The A-bipolar RCT)

Supplement

Caroline Fussing Bruun^1,2^, Helle B. Krogh^6^, Jeff Zarp^1^, Julie Ravneberg Stokholm^1,2^, Julie Lyng Forman^5^, Kamilla Woznica Miskowiak^1,3^, Annamaria Giraldi^6^, Maj Vinberg^2,4^, Maria Faurholt-Jepsen^1,2^, Lars Vedel Kessing^1, 2^

^1^Copenhagen Affective Disorder Research Centre (CADIC), Psychiatric Centre Copenhagen, Copenhagen University Hospital - Mental Health Services CPH, Copenhagen, Denmark

^2^Department of Clinical Medicine, Faculty of Health and Medical Sciences, University of Copenhagen, Copenhagen, Denmark.

^3^Department of Psychology, Faculty of Social Sciences, University of Copenhagen, Copenhagen, Denmark.

^4^The Early Multimodular Prevention and Intervention Research Institution (EMPIRI), Psychiatric Centre North Zealand, Copenhagen University Hospital - Mental Health Services CPH, Hillerød, Denmark;

^5^Section of Biostatistics, Department of Public Health, Faculty of Health and Medical Sciences, University of Copenhagen, Copenhagen, Denmark

^6^ Sexological Clinic, Psychiatric Centre Copenhagen, Copenhagen University Hospital - Mental Health Services CPH, Copenhagen, Denmark

Table of contents

[**Protocol deviations** 3](#_Toc198195290)

[**Inclusion and exclusion criteria** 3](#_Toc198195291)

[**Supplementary Table S1.** **Reasons for non-inclusion of screened patients** 4](#_Toc198195292)

[**Supplementary Tables S2**–**3: Dropouts and exclusions** 5](#_Toc198195293)

[**Supplementary Tables S4–7: Baseline characteristics** 6](#_Toc198195294)

[**Supplementary Tables S8–9: Adherence** 10](#_Toc198195295)

[**Supplementary Figures S1**–**3** 11](#_Toc198195296)

[**Supplementary Table S10: Monthly treatment effect** 14](#_Toc198195297)

[**Supplementary Figures S3–9: Subgroup analyses** 15](#_Toc198195298)

[**Supplementary Tables S11–13:** **Adverse events** 22](#_Toc198195299)

[**Supplementary Tables S14–15: Post-randomization drop-outs** 23](#_Toc198195300)

[**References** 27](#_Toc198195301)

# **Protocol deviations**

**Inclusion criteria:** One participant (female, BD type 1) was 68 years at inclusion; one participant (53-year-old female, BD type 1) was not newly diagnosed

**Tertiary outcomes:** Data for social activity (daily numbers of outgoing and incoming calls, text messages, and time spent on the smartphone) was not available due to regulatory constraints on non-android phones.

**Analyses:** We used an unconstrained LMM for the continuous-time outcomes (mood instability, activity instability, and sleep variability)

# **Inclusion and exclusion criteria**

**Inclusion criteria:** Patients with a primary diagnosis of bipolar disorder (ICD-10 classification), aged 18-65 years, were eligible. The initial diagnostic assessment, with confirmation of an ICD-10 diagnosis of BD and classification into type I or type II according to DSM-IV, was completed by a psychiatrist. Upon inclusion in the study, the diagnosis was confirmed with a Schedules for Clinical Assessment in Neuropsychiatry interview, conducted by trained trial investigators (medical doctors or psychiatrist, PhD).

**Exclusion criteria:** Severe renal disease; severe cardiac insufficiency; a history of gastric ulcer, gastrointestinal bleeding, thrombocytopenia, or other pathological bleeding tendency; concomitant treatment with aspirin, NSAIDs, antithrombotic agents, or SSRIs; and, for female patients, pregnancy, or lactation. Female patients in the childbearing age were required to use contraception during trial participation.

# **Supplementary Table S1.** **Reasons for non-inclusion of screened patients**

| **Reason** | **Frequency** |
| --- | --- |
| Already taking aspirin | 4 |
| Ambivalence towards the diagnosis/treatment | 2 |
| Aspirin contraindicated due to neurological disease | 1 |
| Aspirin hypersensitivity | 1 |
| Diagnostic uncertainty | 5 |
| Fear of side effects | 6 |
| Language barrier | 5 |
| Lost contact after initial screening | 11 |
| No show at enrollment; lost contact | 6 |
| Not motivated | 35 |
| Not possible to get in touch with patient | 16 |
| Ongoing substance abuse | 1 |
| Participation too time consuming | 15 |
| Patient about to move to another region/country | 6 |
| Patient cancelled enrollment visit | 10 |
| Patient disagreed with the design of the RCT | 1 |
| Pregnant | 1 |
| Reluctant to use contraception | 6 |
| Reluctant to use Monsenso | 13 |
| On SSRI | 6 |
| Too demanding to participate | 39 |
| Ulcus | 11 |
| Unknown | 7 |
| Unwilling to take trial medication | 21 |
| Total | 229 |

# **Supplementary Tables S2**–**3: Dropouts and exclusions**

**Supplementary Table S2.** Number of dropouts by allocation group

| **Allocation** | **Dropout before T3** | **Dropout after T3** | **Total** | **Completed T6** |
| --- | --- | --- | --- | --- |
| PBO | 11 | 11 | 22 | 103 |
| LDA | 18 | 10 | 28 | 97 |
| Total sample | 29 | 21 | 50 | 200 |

P-values were calculated using chi-squared test. Total dropouts between groups *p* = 0.429; dropouts stratified by T3 status (comparing dropout rates before or after T3) *p* = 0.467

**Supplementary Table S3.** Reasons for dropout/exclusion after randomization by allocation group

| **Reason, n (%)** | **PBO** (n = 125) | **LDA** (n = 125) | **Total** (n = 250) |
| --- | --- | --- | --- |
| Dizziness | 0 (0) | 1 (0.8) | 1 (0.4) |
| Depression | 1 (0.8) | 1 (0.8) | 2 (0.8) |
| Fear of side effects | 1 (0.8) | 0 (0) | 1 (0.4) |
| Lost contact | 6 (4.8) | 10 (8) | 16 (6.4) |
| Monsenso (pathologized) | 2 (1.6) | 2 (1.6) | 4 (1.6) |
| Not motivated | 3 (2.4) | 1 (0.8) | 4 (1.6) |
| Pregnancy | 2 (1.6) | 2 (1.6) | 4 (1.6) |
| SAE | 1 (0.8) | 0 (0) | 1 (0.4) |
| SAR | 0 (0) | 1 (0.8) | 1 (0.4) |
| Side effect (dyspepsia) | 2 (1.6) | 2 (1.6) | 4 (1.6) |
| Too unstable in BD | 4 (3.2) | 6 (4.8) | 10 (4) |
| Diagnosis revised to UD | 0 (0) | 1 (0.8 | 1 (0.4) |
| Unkown | 0 (0) | 1 (0.8) | 0 (0) |

*Abbreviations*: PBO: Placebo; LDA: Low-Dose Aspirin; BD: Bipolar Disorder; UD: Unipolar Depression

# **Supplementary** **Tables S4–7: Baseline characteristics**

**Supplementary Table S4.** Somatic status at baseline

|  | **Total** (n = 250) | | **PBO** (n = 125) | | **LDA** (n = 125) | |
| --- | --- | --- | --- | --- | --- | --- |
|  | **n** | **%** | **n** | **%** | **n** | **%** |
| Hypothyroidism | 11 | 4.4 | 5 | 4.0 | 6 | 4.8 |
| Hyperthyroidism | 1 | 0.4 | 0 | 0 | 1 | 0.8 |
| Diabetes type 1 | 6 | 2.4 | 3 | 2.4 | 3 | 2.4 |
| Diabetes type 2 | 2 | 0.8 | 2 | 1.6 | 0 | 0 |
| Hypertension | 5 | 2 | 2 | 1.6 | 3 | 2.4 |
| Autoimmune disease | 7 | 2.8 | 4 | 3.2 | 3 | 2.4 |
| Cardiac disease | 2 | 0.8 | 2 | 1.6 | 0 | 0 |
| Dyslipidemia (no statins) | 14 | 5.6 | 10 | 8.0 | 4 | 3.2 |
| Dyslipidemia (**+**statins) | 3 | 1.2 | 1 | 0.8 | 2 | 1.6 |
| Renal disease | 0 | 0 | 0 | 0 | 0 | 0 |
| Neurological disease* | 0 | 0 | 0 | 0 | 0 | 0 |
| Headache <1 day/month | 38 | 15.2 | 18 | 14.4 | 20 | 16.0 |
| Headache ≤14 days/month | 33 | 13.2 | 21 | 16.8 | 12 | 9.6 |
| Headache ≥15 days/month | 14 | 5.6 | 7 | 5.6 | 7 | 5.6 |
| Musculoskeletal pain, monthly | 30 | 12.0 | 18 | 14.4 | 12 | 9.6 |
| Musculoskeletal pain, weekly | 29 | 11.6 | 16 | 12.8 | 13 | 10.4 |
| Musculoskeletal pain, daily | 32 | 12.8 | 14 | 11.2 | 18 | 14.4 |

*Abbreviations*: PBO: Placebo; LDA: Low-Dose Aspirin

*****Excluding migraine, which was registered as ‘headache’

**Supplementary Table S5.** Baseline lifestyle variables

|  | **Total** (n = 250) | | **PBO** (n = 125) | | **LDA** (n = 125) | |
| --- | --- | --- | --- | --- | --- | --- |
| Never smoker (n, %) | 90 | 36.0 | 48 | 38.4 | 42 | 33.6 |
| Ex-smoker (n, %) | 57 | 22.8 | 24 | 19.2 | 33 | 26.4 |
| Current smoker (n, %) | 103 | 41.2 | 53 | 42.4 | 50 | 40.0 |
| If current or ex-smoker: Pack-years (Mdn, Q1; Q3) | 3.0 | 0.8; 7.0 | 3.5 | 0.9; 8.8 | 2.0 | 0.8; 5.2 |
| Alcohol consumption, units/week (Mdn, Q1; Q3) | 3.0 | 1.0; 7.0 | 2.5 | 1.0; 7.0 | 3.0 | 1.0; 8.0 |
| History of harmful use of alcohol*, (n, %) | 32 | 12.8 | 15 | 12% | 17 | 13.6 |
| BMI (Mdn, Q1; Q3) | 23.7 | 21.1; 26.8 | 23.7 | 21.4; 26.7 | 23.5 | 21.1; 26.9 |
| Waist-hip ratio (Mdn, Q1; Q3) | 0.80 | 0.80; 0.90 | 0.80 | 0.90 | 0.80 | 0.90 |

*Abbreviations*: PBO: Placebo; LDA: Low-Dose Aspirin; Mdn: Median; Q1: 25^th^ percentile; Q3: 75^th^ percentile; BMI: Body Mass Index

*Defined as harmful use of alcohol (F10.1) or alcohol dependence syndrome (F10.2)

**Supplementary Table S6a.** Baseline medication

|  | **Total** (n = 250) | | **PBO** (n = 125) | | **LDA** (n = 125) | |
| --- | --- | --- | --- | --- | --- | --- |
| **Medication class** | **n** | **%** | **n** | **%** | **n** | **%** |
| Antidepressants | 11 | 4.4 | 4 | 3.2 | 7 | 5.6 |
| Antipsychotics | 123 | 49.2 | 60 | 48.0 | 63 | 50.4 |
| Antiepileptics (lamotrigene) | 131 | 52.4 | 65 | 52.0 | 66 | 52.8 |
| Lithium | 118 | 47.2 | 57 | 45.6 | 61 | 48.8 |
| Benzodiazepines or Z-hyponotics PN | 17 | 6.8 | 8 | 6.4 | 9 | 7.2 |

**Supplementary Table S6b.** Use of antipsychotics

|  | **Total** (n = 250) | | **PBO** (n = 125) | | **LDA** (n =125) | |
| --- | --- | --- | --- | --- | --- | --- |
| **Antipsychotic** | **n** | **%** | **n** | **%** | **n** | **%** |
| Olanzapine | 4 | 1.6 | 1 | 0.8 | 3 | 2.4 |
| Aripiprazole | 2 | 0.8 | 1 | 0.8 | 1 | 0.8 |
| Lurasidone | 2 | 0.8 | 0 | - | 2 | 1.6 |
| Quetiapine | 112 | 44.8 | 57 | 45.6 | 55 | 44.0 |
| <100 mg daily | 66 | 26.4 | 31 | 24.8 | 35 | 28 |
| ≥100 mg daily | 46 | 18.4 | 26 | 20.8 | 20 | 16.0 |
| Other | 1 | 0.4 | 1 | 0.8 | 0 | 0 |
| Unknown | 2 | 0.8 | 0 | 0 | 2 | 1.6 |

**Supplementary Table S6c.** Lithium and lamotrigine doses

|  | **PBO** | | | **LDA** | | |
| --- | --- | --- | --- | --- | --- | --- |
|  | **n** | **Median** | **Q1; Q3** | **n** | **Median** | **Q1; Q3** |
| Lithium daily dose in mg | 57 | 750 | 450.0; 900 | 61 | 750 | 300; 900 |
| Lamotrigene daily dose in mg | 65 | 200 | 100; 300 | 66 | 200 | 125; 300 |

*Abbreviations*: PBO: Placebo; LDA: Low-Dose Aspirin; Mdn: Median; Q1: 25^th^ percentile; Q3: 75^th^ percentile; PN: pro re nata/as needed

**Supplementary Table S7.** Baseline ratings and questionnaire scores

|  | **Total** | | | **PBO** | | | **LDA** | | |
| --- | --- | --- | --- | --- | --- | --- | --- | --- | --- |
| **Ratings** | **n** | **Mean** | **SD** | **n** | **Mean** | **SD** | **n** | **Mean** | **SD** |
| YMRS | 250 | 5.2 | 4.7 | 125 | 5.0 | 4.6 | 125 | 5.4 | 4.7 |
| HDRS-6 | 250 | 5.0 | 3.6 | 125 | 4.8 | 3.7 | 125 | 5.2 | 3.5 |
| HDRS-17 | 250 | 9.8 | 6.1 | 125 | 9.2 | 6.2 | 125 | 10.3 | 6.0 |
| **Functional capacity** | **n** | **Mean** | **SD** | **n** | **Mean** | **SD** | **n** | **Mean** | **SD** |
| FAST total score | 250 | 18.7 | 11.8 | 125 | 18.4 | 11.9 | 125 | 19.1 | 11.7 |
| **Cognition** | **n** | **Mean** | **SD** | **n** | **Mean** | **SD** | **n** | **Mean** | **SD** |
| SCIP total score | 241 | 77.7 | 10.5 | 121 | 78.1 | 11.0 | 120 | 77.2 | 9.9 |
| **Questionnaire scores** | **n** | **Mean** | **SD** | **n** | **Mean** | **SD** | **n** | **Mean** | **SD** |
| Childhood trauma (CTQ, total score) | 238 | 42.9 | 14.2 | 120 | 42.4 | 14.4 | 118 | 43.5 | 14.0 |
| Physical activity (IPAQ, MET-min/week) | 237 | 11672 | 128461 | 120 | 19633 | 180495 | 117 | 3507 | 4458 |
| Sleep quality (PSQI, total score) | 237 | 9.0 | 3.7 | 120 | 8.7 | 3.5 | 117 | 9.2 | 3.8 |
|  | **n** | **Mdn** | **Q1, Q3** | **n** | **Mdn** | **Q1, Q3** | **n** | **Mdn** | **Q1, Q3** |
| Stress (PSS, total score) | 238 | 22 | 18, 25 | 120 | 21 | 17.75, 24 | 118 | 22 | 18, 26 |
| **WHO QoL-BREF** | **n** | **Mean** | **SD** | **n** | **Mean** | **SD** | **N** | **Mean** | **SD** |
| Overall QoL | 239 | 3.4 | 0.9 | 120 | 3.5 | 0.9 | 119 | 3.3 | 0.8 |
| General Health | 239 | 3.0 | 1.0 | 120 | 3.1 | 1.0 | 119 | 3.0 | 0.9 |
| Physical Health | 239 | 48.1 | 12.5 | 120 | 48.3 | 12.7 | 119 | 47.9 | 12.4 |
| Psychological Health | 239 | 49.1 | 14.5 | 120 | 49.4 | 14.3 | 119 | 48.8 | 14.7 |
| Social Relationships | 239 | 61.5 | 18.6 | 120 | 61.7 | 19.5 | 119 | 61.3 | 17.6 |
| Environment | 239 | 63.3 | 12.8 | 120 | 63.2 | 13.5 | 119 | 63.4 | 12.1 |

*Abbreviations*: : PBO: Placebo; LDA: Low-Dose Aspirin; Mdn: Median; Q1: 25^th^ percentile; Q3: 75^th^ percentile; M: mean; SD, standard deviation; YMRS: Young Mania Rating Scale; HDRS-6: Hamilton Depression Rating Scale 6-item version; HDRS-17: Hamilton Depression Rating Scale 17-item version; FAST: Functioning Assessment Short Test; SCIP: Screen for Cognitive Impairment in Psychiatry; CTQ: *Abbreviations*: PBO: Placebo; LDA: Low-Dose Aspirin; PSS: Cohen’s Perceived Stress Scale; IPAQ: International Physical Activity Questionnaire; MET, Metabolic Equivalent Task; PSQI: Pittsburgh Sleep Quality Index; WHO QoL-BREF: WHO Quality of life-BREF;

# **Supplementary Tables S8–9: Adherence**

**Supplementary Table S8.** Self-reported adherence to trial medication: Number of patients taking the medication ≥ 50% of days

| **Visit** | **PBO – n (%)** | **LDA – n (%)** |
| --- | --- | --- |
| 3-month follow-up (PBO: n = 113; LDA: n = 107) | 108 (96) | 98 (92) |
| 6-month follow-up (PBO: n = 103; LDA: n = 97) | 100 (97) | 92 (95) |
| 12-month follow-up (PBO: n = 42; LDA: n = 41) | 41 (98) | 36 (88) |

**Supplementary Table S9**. Self-reported reasons for missingness of mood ratings

|  | **PBO** (n = 69) | | **LDA** (n = 71) | | **Total** (n = 140) | |
| --- | --- | --- | --- | --- | --- | --- |
|  | **n** | **%** | **n** | **%** | **n** | **%** |
| Depressive symptoms | 21 | 30.4 | 21 | 29.6 | 42 | 30 |
| Hypomanic/manic symptoms | 5 | 7.2 | 6 | 8.5 | 11 | 7.9 |
| Euthymia/stability | 4 | 5.8 | 2 | 2.8 | 6 | 4.3 |
| Mixed state symptoms | 1 | 1.4 | 1 | 1.4 | 2 | 1.4 |
| Reasons unrelated to BD | 27 | 39.1 | 29 | 40.8 | 56 | 40 |
| Feels worse when attentive to the mood | 1 | 1.4 | 0 | 0 | 1 | 0.7 |
| Other reasons | 10 | 14.5 | 12 | 16.9 | 22 | 15.7 |

*Abbreviations*: PBO: Placebo; LDA: Low-Dose Aspirin; Mdn: Median; Q1: 25^th^ percentile; Q3: 75^th^ percentile; M: mean; SD, standard deviation

# **Supplementary Figures S1**–**3**

**Supplementary Figure S1**. Thromboxane B_2_ concentrations by allocation group

**
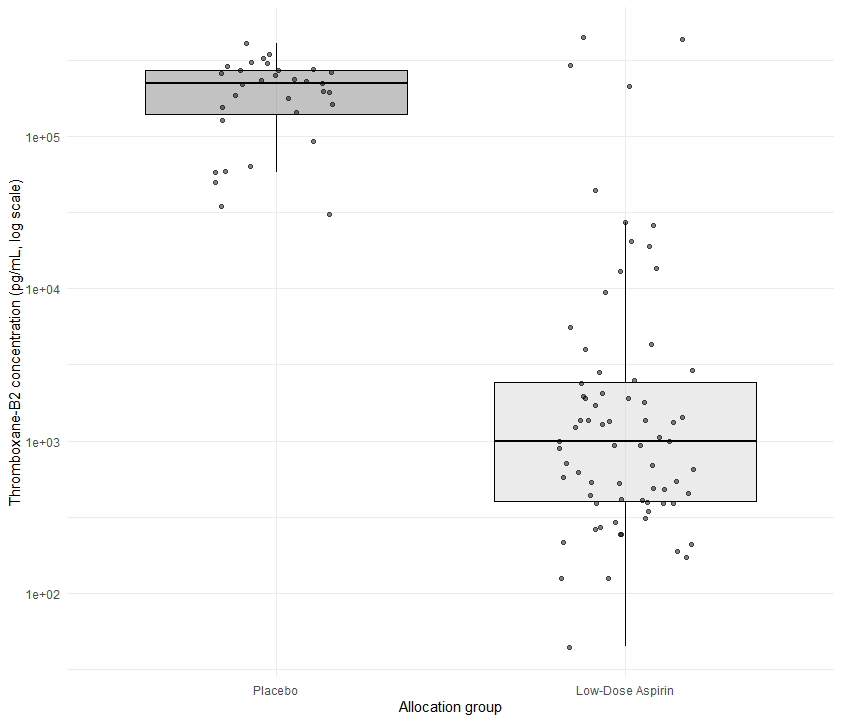
**

Points are individual participant-level data. Each box represents the distribution of thromboxane B_2_ concentrations for participants in each allocation group. Horizontal lines are the is the median (50th percentile); top and bottom edges of the box represent the 25th percentile (Q1) and 75th percentile (Q3).

**Supplementary Figure S2**. Days with mood ratings by allocation group


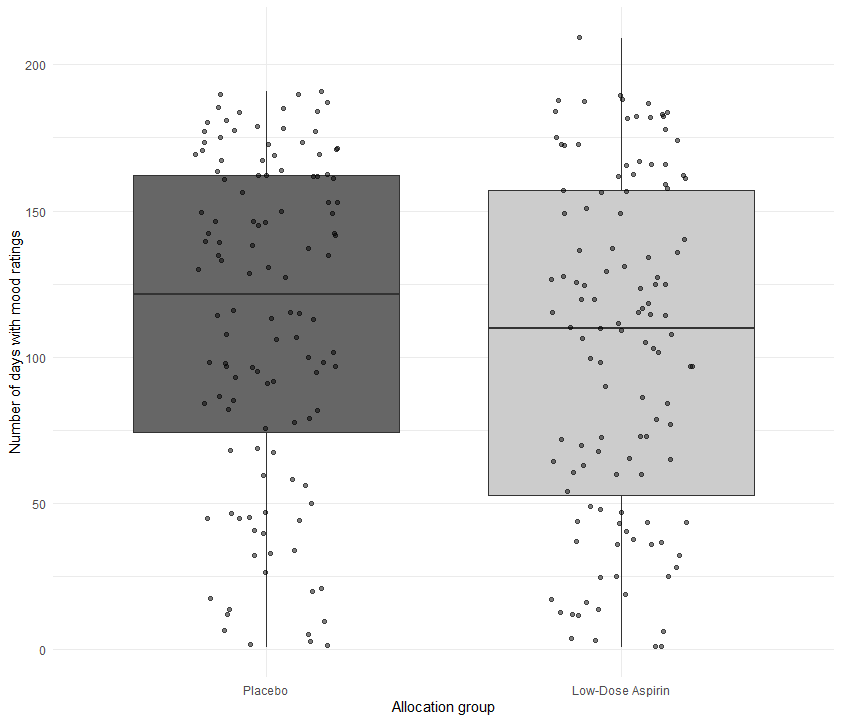


Points are individual participant-level data. Each box represents the distribution of the number of days with mood ratings for participants in each allocation group. Horizontal lines are the is the median (50th percentile); top and bottom edges of the box represent the 25th percentile (Q1) and 75th percentile (Q3).

**Supplementary Figure S3.** Mean monthly mood instability with 95% confidence intervals in patients with bipolar disorder receiving low-dose aspirin


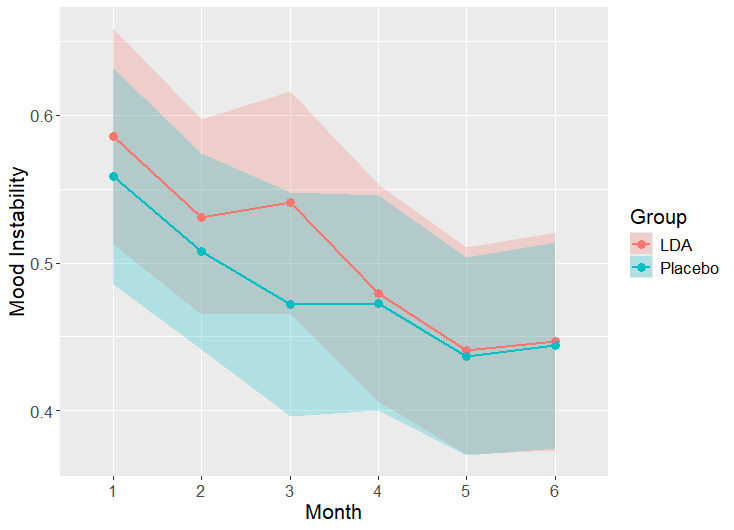


# **Supplementary Table S10: Monthly treatment effect**

**Supplementary Table S10.** Average treatment differences in MI between LDA and placebo reported for each month of the 6-month follow-up, relative to placebo group

| **Month** | **ETD** | **95% CI** | | ***p*-value** |
| --- | --- | --- | --- | --- |
| 1 | 0.027 | −0.077 | 0.13 | 0.610 |
| 2 | 0.023 | −0.0716 | 0.1175 | 0.629 |
| 3 | 0.069 | −0.038 | 0.176 | 0.204 |
| 4 | 0.007 | −0.097 | 0.11 | 0.898 |
| 5 | 0.003 | −0.094 | 0.101 | 0.943 |
| 6 | 0.003 | −0.098 | 0.105 | 0.951 |

*Abbreviations:* ETD: Estimated Treatment Difference, relative to placebo group; 95% CI: 95% Confidence Interval

#

# **Supplementary Figures S3–9: Subgroup analyses**

**Supplementary Figure S3.** Results of subgroup linear mixed model analyses for mood instability from baseline to 6-month follow-up

*Abbreviations:* ETD: Estimated Treatment Difference; 95% CI: 95% Confidence Interval; Mdn: median; BL: baseline; HDRS-17: Hamilton Depression Rating Scale, 17 item version; HDRS-6: Hamilton Depression Rating Scale, 6 item version; YMRS: Young Mania Rating Scale; FDR: First-Degree Relative; dx: diagnosis

**Supplementary Figure S4.** Results of subgroup linear mixed model analyses for mood instability from baseline to 12-month follow-up

*Abbreviations:* ETD: Estimated Treatment Difference; 95% CI: 95% Confidence Interval; Mdn: median; BL: baseline; HDRS-17: Hamilton Depression Rating Scale, 17 item version; HDRS-6: Hamilton Depression Rating Scale, 6 item version; YMRS: Young Mania Rating Scale; FDR: First-Degree Relative; dx: diagnosis

**Supplementary Figure S5.** Results of subgroup linear mixed model analyses for activity instability from baseline to 6-month follow-up

*Abbreviations:* ETD: Estimated Treatment Difference; 95% CI: 95% Confidence Interval; Mdn: median; BL: baseline; HDRS-17: Hamilton Depression Rating Scale, 17 item version; HDRS-6: Hamilton Depression Rating Scale, 6 item version; YMRS: Young Mania Rating Scale; FDR: First-Degree Relative; dx: diagnosis

**Supplementary Figure S6.** Results of subgroup linear mixed model analyses for activity instability from baseline to 12-month follow-up

*Abbreviations:* ETD: Estimated Treatment Difference; 95% CI: 95% Confidence Interval; Mdn: median; BL: baseline; HDRS-17: Hamilton Depression Rating Scale, 17 item version; HDRS-6: Hamilton Depression Rating Scale, 6 item version; YMRS: Young Mania Rating Scale; FDR: First-Degree Relative; dx: diagnosis

**Supplementary Figure S7.** Results of subgroup linear mixed model analyses for depressive symptoms at 6-month follow-up

*Abbreviations:* ETD: Estimated Treatment Difference; 95% CI: 95% Confidence Interval; Mdn: median; BL: baseline; HDRS-17: Hamilton Depression Rating Scale, 17 item version; HDRS-6: Hamilton Depression Rating Scale, 6 item version; YMRS: Young Mania Rating Scale; FDR: First-Degree Relative; dx: diagnosis

**Supplementary Figure S8.** Results of subgroup linear mixed model analyses for depressive symptoms at 12-month follow-up

*Abbreviations:* ETD: Estimated Treatment Difference; 95% CI: 95% Confidence Interval; Mdn: median; BL: baseline; HDRS-17: Hamilton Depression Rating Scale, 17 item version; HDRS-6: Hamilton Depression Rating Scale, 6 item version; YMRS: Young Mania Rating Scale; FDR: First-Degree Relative; dx: diagnosis

**Supplementary Figure S9.** Results of subgroup linear mixed model analyses for manic symptoms at 6 and 12-month follow-up

**

*Abbreviations:* FU: Follow-up; ETD: Estimated Treatment Difference; 95% CI: 95% Confidence Interval; Mdn: median; BL: baseline; YMRS: Young Mania Rating Scale

## **Supplementary Tables S11–13: Adverse events**

Events were classified according to the International Council for Harmonisation of Technical Requirements for Pharmaceuticals for Human Use (ICH) Good Clinical Practice (GCP) guidelines (1).

**Supplementary Table S11:** Summary table of all events by allocation group

| **Event type, n** | **PBO** | **LDA** | ***p*-value** |
| --- | --- | --- | --- |
| Adverse Events | 32 | 39 | 0.400 |
| Adverse Reactions | 6 | 9 | 0.594 |
| Serious Adverse Events | 12 | 13 | 1.000 |
| Serious Adverse Reactions | 0 | 1 | 1.000 |
| All events | 51 | 63 | 0.162 |

**Supplementary Table S12:** Adverse reactions by allocation group

| **Organ system (symptom), n (%)** | **PBO** | **LDA** | ***p*-value** |
| --- | --- | --- | --- |
| Coagulation (Increased bleeding tendency/bruising) | 1 (0.8) | 3 (2.4) | 0.614 |
| Gastrointestinal (Nausea or vomiting) | 0 (0) | 2 (1.6) | 0.478 |
| Gastrointestinal (reflux or pyrosis) | 4 (3.2) | 4 (3.2) | 1.000 |
| Nervous system (headache) | 1 (0.8) | 0 (0) | 1.000 |
| Total | 6 (4.8) | 9 (7.2) | 0.594 |

**Supplementary Table S13:** Serious adverse events by allocation group

| **SAE, n** | **PBO** | **LDA** | ***p*-value** |
| --- | --- | --- | --- |
| Hospitalization (depression) | 10 | 9 |  |
| Hospitalization (mania) | 0 | 1 |  |
| Hospitalization (mixed) | 1 | 1 |  |
| Suicide attempt | 1 | 0 |  |
| Suicide | 0 | 1 |  |
| Ketoacidosis obs pro (in participant with type 1 diabetes) | 0 | 1 |  |
| Total | 12 | 13 | 1.000 |

P-values were calculated using Chi-squared test or Fisher's Exact Test, as appropriate

## **Supplementary Tables S14–15: Post-randomization drop-outs**

**Supplementary Table S14.** Characteristics of completers and non-completers, with completers defined as participants that completed the 6-month follow-up visit

|  | **Completers** (n = 200) | | **Non-completers** (n = 50) | | ***p*-value** |
| --- | --- | --- | --- | --- | --- |
|  | **PBO** (n = 103) | **LDA** (n = 97) | **PBO** (n = 22) | **LDA** (n = 28) |  |
| **Demographics** |  |  |  |  |  |
| Age, Mdn [Q1; Q3] | 28 [24.5; 39] | 30 [25; 37] | 32.5 [24.5; 39] | 27.5 [25; 35.25] | 0.176 |
| Female sex, n (%) | 60 (58.3) | 58 (59.8) | 12 (54.5) | 15 (53.6) | 0.927 |
| Married/registered partnership or stable relationship, n (%) | 54 (52.4) | 46 (47.9) | 15 (68.2) | 11 (39.3) | 0.206 |
| Years of education*, M (SD) | 14.5 (2.6) | 14.9 (2.6) | 16 (2.9) | 14.8 (1.9) | 0.118 |
| Occupation (%) |  |  |  |  | 0.216 |
| - *Full time employed* | 27(26.2) | 22(22.7) | 4(18.2) | 6(21.4) |  |
| - *Part time employed* | 4(3.9) | 10(10.3) | 1(4.5) | 0(0) |  |
| - *Other (hourly based, free-lance, etc.)* | 5(4.9) | 1(1.0) | 1(4.5) | 2(7.1) |  |
| - *Student* | 35(34.0) | 34(35.1) | 7(31.8) | 7(25.0) |  |
| - *Stay-at-home* | 0(0) | 1(1.0) | 0(0) | 0(0) |  |
| - *Retiree* | 2(1.9) | 3(3.1) | 0(0) | 0(0) |  |
| - *Conscript* | 0(0) | 1(1.0) | 0(0) | 0(0) |  |
| - *Long-term sickleave* | 18(17.5) | 14(14.4) | 8(36.4%) | 6(21.4) |  |
| - *Unemployed* | 8 (7.8) | 8 (8.2) | 1(4.6) | 4(14.3) |  |
| - *Transfer income* | 4(3.8) | 3(3.2) | 0 (0) | 3(10.8) |  |
| Longterm sickleaves** due to BD, Mdn [Q1; Q2] | 2 [1; 5] | 1 [1; 3] | 2.5 [1; 4] | 2 [1; 3] | 0.749 |
| **Clinical characteristics** |  |  |  |  |  |
| Bipolar type I, n (%) | 54 (52.4) | 42 (43.3) | 11 (50) | 13 (46.4) | 0.630 |
| Age at illness onset, Mdn [Q1; Q3] | 17 [15; 20] | 19 [16; 23] | 18 [15.25; 20] | 18 [17; 21.25] | 0.028 |
| Illness duration (years), Mdn [Q1; Q3] | 10 [6; 21] | 10 [5; 16] | 15.5 [10.25; 19.5] | 10.5 [6; 16.75] | 0.311 |
| Index episode, n (%) |  |  |  |  | 0.962 |
| - - - *Depression* | 84 (81.6) | 78 (82.1) | 17 (77.3) | 23 (82.1) |  |
| - - - *Mania* | 4 (3.9) | 3 (3.2) | 1 (4.5) | 2 (7.1) |  |
| - - - *Hypomania* | 15 (14.6) | 13 (13.7) | 4 (18.2) | 3 (10.7) |  |
| - - - *Mixed* | 0 (0) | 1 (1.1) | 0 (0) | 0 (0) |  |
| - - - *Unknown* | 0 (0) | 2 | 0 (0) | 0 (0) |  |
| Depressive episodes, Mdn [Q1; Q3] | 8 [5; 20] | 7 [4; 14.25] | 6 [4.25; 14.25] | 10 [4; 19.5] | 0.491 |
| Hypomanic episodes, Mdn [Q1; Q3] | 8 [4; 20] | 5 [3; 15] | 7.5 [2.25; 15.5] | 10 [4; 18.5] | 0.196 |
| Manic episodes, Mdn [Q1; Q3] | 0 [0; 2] | 0 [0; 1] | 0 [0; 2] | 0 [0; 2] | 0.197 |
| Mixed episodes, M (SD) | 0 [0; 1] | 0 [0; 0] | 0 [0; 1] | 0 [0; 1] |  |
| ≥1 suicide attempt(s), n (%) | 23 (22) | 15 (15) | 5 (23) | 7 (25) | 0.545 |
| ≥1 hospitalization(s), n (%) | 42 (41) | 31 (32) | 9 (41) | 8 (29) | 0.942 |
| ≥1 psychotic episode, n (%) | 31 (30) | 22 (23) | 5 (23) | 11 (39) | 0.421 |
| HDRS-17 total score, Mdn [Q1; Q3] | 8 [4; 13] | 9 [7; 14] | 8.5 [6; 12.8] | 8.5 [5; 13.2] | 0.359 |
| HDRS-6 total score, Mdn [Q1; Q3] | 4 [2; 7.5] | 5 [3; 8] | 4.5 [2.2; 7] | 4.5 [1.8; 7] | 0.563 |
| YMRS total score, Mdn [Q1; Q3] | 4 [2; 7] | 4 [2; 7] | 4 [2; 7.5] | 5.5 [3; 8.8] | 0.306 |
| **Psychotropic medication, n (%)** |  |  |  |  |  |
| Antidepressants | 5 | 4 | 0 (0) | 2 | 0.710 |
| Antipsychotics | 53 | 49 | 7 | 14 | 0.400 |
| Lamotrigene | 54 | 51 | 11 | 15 | 0.995 |
| Lithium | 49 | 46 | 8 | 15 | 0.682 |
| Benzodiazepines or Z-hypnotics PN | 7 | 9 | 1 | 0 (0) | 0.572 |
| **Functional capacity** |  |  |  |  |  |
| FAST total score, Mdn [Q1; Q3] | 16 [8.5; 24] | 16 [10; 26] | 20.5 [13.2; 28.8] | 18.5 [12.5; 32.2] | 0.384 |
| **Cognition** |  |  |  |  |  |
| SCIP total score, Mdn [Q1; Q3] | 80 [71; 88] | 77 [72; 84] | 76 [67; 82] | 76 [70.5; 83] | 0.166 |

*Abbreviations*: Mdn: Median; Q1: 25^th^ percentile; Q3: 75^th^ percentile; M: mean; SD, standard deviation; ICD: International Classification of Diseases; HDRS-6: Hamilton Depression Rating Scale 6-item version; HDRS-17: Hamilton Depression Rating Scale 17-item version; YMRS: Young Mania Rating Scale; SSRIs: Selective Serotonin Reuptake Inhibitors; PN: pro re nata/as needed; FAST: Functioning Assessment Short Test; SCIP: Screen for Cognitive Impairment in Psychiatry; MET: Metabolic Equivalent Task

*Completed years, starting with primary school; **Defined as sick leave ≥2 weeks

P-values were calculated using chi-squared or Fisher's exact tests for categorical variables and t-tests or Wilcoxon rank-sum tests for continuous variables, as appropriate.

**Supplementary Table S15.** Characteristics of participants who were per-protocol and non-adherent, with per-protocol prespecified as participants completing ≥ 30 days of mood assessments and ≥ 90 days of self-reported compliance to mediation

|  | **Per-protocol** (n =157) | | **Non-adherent** (n = 93) | | ***p*-value** |
| --- | --- | --- | --- | --- | --- |
|  | **PBO** (n = 80) | **LDA** (n = 77) | **PBO** (n = 45) | **LDA** (n = 48) |  |
| **Demographics** |  |  |  |  |  |
| Age, Mdn [Q1; Q3] | 31 [26; 44.25] | 30 [26; 38] | 28 [24; 37] | 28 [25; 35.25] | 0.237 |
| Female sex, n (%) | 46 (57.5) | 47 (61.0) | 26 (57.8) | 26 (54.2) | 0.899 |
| Married/registered partnership or stable relationship, n (%) | 42 (52.5) | 35 (46.1) | 27 (60.0) | 22 (45.8) | 0.428 |
| Years of education*, M (SD) | 14.84 (2.63) | 14.77 (2.66) | 14.73 (2.92) | 15.12 (2.10) | 0.871 |
| Occupation, n (%) |  |  |  |  | 0.454 |
| - *Full time employed* | 19(23.8) | 16 (20.8) | 12(26.7) | 12 (25.0) |  |
| - *Part time employed* | 2 (2.5) | 9 (11.7) | 3 (6.7) | 1 (2.1) |  |
| - *Other (hourly based, free-lance, etc.)* | 4 (5.0) | 1 (1.3) | 2 (4.4) | 2 (4.2) |  |
| - *Student* | 27 (33.8) | 25 (32.5) | 15 (33.3) | 16 (33.3) |  |
| - *Stay-at-home* | 0 (0) | 1 (1.3) | 0 (0) | 0 (0) |  |
| - *Retiree* | 2 (2.5) | 3 (3.9) | 0 (0) | 0 (0) |  |
| - *Conscript* | 0 (0) | 0 (0) | 0 (0) | 1 (2.1) |  |
| - *Long-term sickleave* | 18 (22.5) | 12 (15.6) | 8 (17.8) | 8 (16.7) |  |
| - *Unemployed* | 7 (8.8) | 6 (7.8) | 2 (4.4) | 6 (12.5) |  |
| - *Transfer income* | 1 (1.1) | 4 (5.1) | 3(6.7) | 2 (4.1) |  |
| Longterm sickleaves** due to BD, Mdn [Q1, Q3] | 2 [1; 5] | 2 [1; 3] | 2 [1; 4] | 2 [1; 3] | 0.481 |
| **Clinical characteristics** |  |  |  |  |  |
| Bipolar type I (n, %) | 37 (46.2) | 32 (41.6) | 28 (62.2) | 23 (47.9) | 0.170 |
| Age at illness onset, Mdn [Q1, Q3] | 17.5 [15; 21] | 19 [17; 22] | 17 [15; 19] | 18.5 [15.75; 23] | 0.029 |
| Illness duration (years), Mdn [Q1, Q3] | 11 [6; 24.25] | 11 [5; 18] | 11 [6; 16] | 9.5 [5; 13.25] | 0.426 |
| Index episode (n, %) |  |  |  |  | 0.927 |
| - - - *Depression* | 64 (80.0) | 63 (82.9) | 37 (82.2) | 38 (80.9) |  |
| - - - *Mania* | 3 (3.8) | 2 (2.6) | 2 (4.4) | 3 (6.4) |  |
| - - - *Hypomania* | 13 (16.2) | 10 (13.2) | 6 (13.3) | 6 (23.8) |  |
| - - - *Mixed* | 0 (0) | 1 (1.3) | 0 (0) | 0 (0) |  |
| - - - *Unknown* | 0 (0) | 1 (1.3) | 0 (0) | 0 (0) |  |
| Depressive episodes, Mdn [Q1, Q3] | 8 [5; 20] | 7.5 [4; 18.5] | 7 [4.75; 12.5] | 7 [4; 14.5] | 0.546 |
| Hypomanic episodes | 8 [3.5; 20] | 6 [3; 20] | 10 [3; 20] | 5.5 [3; 10.75] | 0.623 |
| Manic episodes, Mdn [Q1, Q3] | 0 [0; 2] | 0 [0; 1] | 0 [0; 2] | 0 [0; 2] | 0.070 |
| Mixed episodes, M (SD) | 0 [0; 0] | 0 [0; 0] | 0 [0; 1] | 0 [0; 0.75] | 0.548 |
| ≥1 suicide attempt(s), n (%) | 16 (20.0) | 14 (18.2) | 12 (26.7) | 8 (16.7) | 0.628 |
| ≥1 hospitalization(s), n (%) | 28 (35.0) | 28 (36.4) | 23 (51.1) | 11 (22.9) | 0.045 |
| ≥1 psychotic episode, n (%) | 21 (26.3) | 15 (19.5) | 15 (33.3) | 18 37.5) | 0.085 |
| HDRS-17 total score, Mdn [Q1; Q3] | 9 [4; 12.2] | 9 [7; 15] | 7 [5; 13] | 8.5 [5; 13] | 0.258 |
| HDRS-6 total score, Mdn [Q1; Q3] | 5 [2; 8] | 5 [3; 8] | 4 [2; 7] | 4 [2; 7] | 0.271 |
| YMRS total score, Mdn [Q1; Q3] | 3 [2; 7] | 4 [2; 7] | 4 [2; 8] | 4 [2; 9.5] | 0.526 |
| **Psychotropic medication, n (%)** |  |  |  |  |  |
| Antidepressants (non-SSRIs) | 2 (2.5) | 4 (5.2%) | 2 (4.4) | 3 (6.3) | 0.674 |
| Antipsychotics | 41 (51.3) | 41 (53.2) | 19 (42.2) | 22 (45.8) | 0.630 |
| Lamotrigene | 43 (53.8) | 42 (54.5) | 22 (48.9) | 24 (50.0) | 0.911 |
| Lithium | 37 (46.3) | 35 (45.5) | 20 (44.4) | 26 (54.2%) | 0.754 |
| Benzodiazepines or Z-hypnotics PN | 6 (7.5) | 8 (10.4) | 2 (4.4) | 1 (2.1) | 0.411 |
| **Functioning** |  |  |  |  |  |
| FAST total score, Mdn [Q1; Q3] | 17 [10; 24] | 16 [10; 28] | 17 [9; 25] | 16 [9; 27.5] | 0.981 |
| **Cognition** |  |  |  |  |  |
| SCIP total score, M (SD) | 80.5 [70.2; 87.8] | 77.5 [71.1; 83.8] | 77 [71; 86.5] | 77 [71.2; 83.8] | 0.756 |

*Abbreviations*: Mdn: Median; Q1: 25^th^ percentile; Q3: 75^th^ percentile; M: mean; SD, standard deviation; ICD: International Classification of Diseases; HDRS-6: Hamilton Depression Rating Scale 6-item version; HDRS-17: Hamilton Depression Rating Scale 17-item version; YMRS: Young Mania Rating Scale; SSRIs: Selective Serotonin Reuptake Inhibitors; PN: pro re nata/as needed; FAST: Functioning Assessment Short Test; SCIP: Screen for Cognitive Impairment in Psychiatry; MET: Metabolic Equivalent Task

*Completed years, starting with primary school; **Defined as sick leave ≥2 weeks

P-values were calculated using chi-squared or Fisher's exact tests for categorical variables and t-tests or Wilcoxon rank-sum tests for continuous variables, as appropriate.

## **References**

1. International Council for Harmonisation of Technical Requirements for Pharmaceuticals for Human Use (ICH). ICH Harmonised Guideline: Integrated Addendum to ICH E6(R1): Guideline for Good Clinical Practice E6(R2). Geneva. Switzerland; 2016.
